# Comparisons of deep learning algorithms for diagnosing bacterial keratitis via external eye photographs

**DOI:** 10.1038/s41598-021-03572-6

**Published:** 2021-12-20

**Authors:** Ming-Tse Kuo, Benny Wei-Yun Hsu, Yi-Sheng Lin, Po-Chiung Fang, Hun-Ju Yu, Alexander Chen, Meng-Shan Yu, Vincent S. Tseng

**Affiliations:** 1 Department of Ophthalmology, Kaohsiung Chang Gung Memorial Hospital and Chang Gung University College of Medicine, No.123, Dapi Rd., Niaosong Dist., Kaohsiung City, 833 Taiwan (R.O.C.); 2 School of Medicine, Chang Gung University, Taoyuan City, 33302 Taiwan; 3 Department of Computer Science, National Yang Ming Chiao Tung University, No. 1001, Daxue Rd., East Dist., Hsinchu City, 300 Taiwan (R.O.C.)

**Keywords:** Preclinical research, Translational research

## Abstract

Bacterial keratitis (BK), a painful and fulminant bacterial infection of the cornea, is the most common type of vision-threatening infectious keratitis (IK). A rapid clinical diagnosis by an ophthalmologist may often help prevent BK patients from progression to corneal melting or even perforation, but many rural areas cannot afford an ophthalmologist. Thanks to the rapid development of deep learning (DL) algorithms, artificial intelligence via image could provide an immediate screening and recommendation for patients with red and painful eyes. Therefore, this study aims to elucidate the potentials of different DL algorithms for diagnosing BK via external eye photos. External eye photos of clinically suspected IK were consecutively collected from five referral centers. The candidate DL frameworks, including ResNet50, ResNeXt50, DenseNet121, SE-ResNet50, EfficientNets B0, B1, B2, and B3, were trained to recognize BK from the photo toward the target with the greatest area under the receiver operating characteristic curve (AUROC). Via five-cross validation, EfficientNet B3 showed the most excellent average AUROC, in which the average percentage of sensitivity, specificity, positive predictive value, and negative predictive value was 74, 64, 77, and 61. There was no statistical difference in diagnostic accuracy and AUROC between any two of these DL frameworks. The diagnostic accuracy of these models (ranged from 69 to 72%) is comparable to that of the ophthalmologist (66% to 74%). Therefore, all these models are promising tools for diagnosing BK in first-line medical care units without ophthalmologists.

## Introduction

Infectious keratitis (IK) is a severe corneal infection that is cartegorized into viral keratitis (VK), bacterial keratitis (BK), fungal keratitis (FK), and parasitic keratitis (PK)^1^. BK is one of the most common and vision-threatening types of IK^2,3^. The most common risk factor for BK is contact lens wear, which has a growing popularity worldwide due to various purposes such as exercise, cosmesis, and myopic control^4^. Compared to other IKs, BK is much more fulminant and painful in the clinical course. A delayed diagnosis of BK has the potential to lead to large-area corneal ulcerations, melting, and even perforation. Thus, prompt diagnosis and treatment of BK are critical objectives in the face of IK. However, the ophthalmologist supply in many rural settings does not meet the demand for the desired speed of diagnosing BK.

Convolutional Neural Network (CNN) has been demonstrated to be highly effective in employing deep learning (DL) on classifying images^5,6^. Following the fast development of DL algorithms, artificial intelligence (AI) via image recognition could provide eye-pain patients with a primary screening of BK. Several extremely efficient DL algorithms, including ResNet^7^, DenseNet^8^, ResNeXt^9^, SENet^10^, and EfficientNet^11^, have the potential to develop a model for image diagnosis of BK, and have been demonstrated to be effective in several medical applications^12–14^.

ResNet brought a breakthrough in deep CNN for image processing^7^. It proposed the residual block, which can be seen as a set of layers. Inside each block, additional connections skip one or more layers like shortcuts that perform identity transformation. The residual block design helped make the network structure deeper without facing the degradation problem, which has been observed that deeper structures lead to higher errors in the training process than saturated ones. DenseNet is a representative CNN-based method with fewer computations and is more effective than ResNet^8^. In DenseNet, the dense blocks can be thought of as an enhanced version of the residual block. Instead of one shortcut in each block, it connects all layers directly with each other inside the block in a feedforward manner. Moreover, DenseNet combines the feature maps learned by different layers with concatenation, increasing the input variation of subsequent layers and ameliorating efficiency.

ResNeXt was proposed based on the concept of ResNet^9^. It exploited a split-transform-merge strategy, splitting a module block into multi-branch low-dimensional embeddings to perform transformation and aggregated by summation as output. For comparison, the shortcut connection in ResNet can be taken as a two-branch network where one branch is the identity mapping. This strategy exposes a new factor, cardinality, which impacts the dimension of depth and width and supports building an effective multi-branch structure while maintaining computation complexity. SENet used the channel-attention idea to make DL models learn the crucial channels in the training process. The channel-attention is a concept that the model considers the relationship between each channel inside the CNN structure and gives greater attention or heavier weights on crucial channels learned from the training process. Moreover, it can be applied to many existing DL methods to boost their performance, such as SE-ResNet^10^. EfficientNet was developed by a technique of neural architecture search, utilizing the search approach for a baseline neural architecture (EfficientNet B0) optimized with both accuracy and computation cost. After that, the baseline network was scaled to generate other EfficientNets (from B1 and up to B7) by a compound scaling method that used a compound coefficient to uniformly scale the network depth, width, and resolution to get a better performance^11^.

Recently, two researches demonstrated a DL model via external eye photos with a terrific performance in diagnosing BK^15,16^. However, one adopted two kinds of images (external eye photos and fluorescence staining photos) and processed these photos with an image segmentation technique^16^. The other adopted a specific image transformation technique before running a DL diagnostic model^15^. In this study, we aimed to elucidate the faithful performance of different DL models in diagnosing BK via an external eye photo. Thus, this study compared the presentation of DL models based on image level and used a single external eye photo without other preprocessing techniques, such as image transformation or segmentation.

## Materials and methods

### Study design & subjects

We collected external eye photos and reviewed medical records from patients with clinically suspected IK who presented to five Chang Gung Memorial Hospital (CGMH) branches from June 1, 2007 to May 31, 2019. According to the individual standard procedures in CGMH branches, external eye photography was performed by certified ophthalmic technicians using a camera-mounted slit lamp biomicroscope. One photo using white light illumination (no enhancing slit beam) was collected for each patient in the following experiments. The study was approved by the Chang Gung Medical Foundation Institutional Review Board (Ethical approval code: 201901255B0C601) and adhered to the ARVO statement on human subjects and the Declaration of Helsinki. The Chang Gung Medical Foundation Institutional Review Board waived the need for informed consent for patients in this study based on a retrospective design and the privacy protection via delinking personal identification at image and data analysis.

The definition of IK from the enrolled patients must meet one of the following criteria: (1) at least one of the following laboratory confirmations, including direct microscopy (Gram or acid-fast stain), culture (blood agar, chocolate agar, Sabouraud dextrose agar, or Löwenstein–Jensen slant) and molecular tests (polymerase chain reaction, or dot hybridization assay) for corneal scraping samples, and pathological examination for corneal biopsy samples^17–20^, (2) three experienced corneal specialists (≥ 8 years of qualification in the specialty) made a consensus impression of one specific kind of IK for the same case. The subject was excluded if (1) mixed infections or contaminated organisms such as *Staphylococcus epidermidis* or *Micrococcus* spp. were reported by laboratory tests and (2) three experienced corneal specialists could not reach a consensus impression. Via disease code tracking, a total of 1985 photos from 1985 clinically suspected IK patients were initially included, while only 1512 photos from 1512 patients were enrolled after exclusion.

### Image preprocessing of subjects’ external eye photos

The procedure of image preprocessing was similar to our previous report^21^. In brief, the date of photography and identification information footnoted in the photo were pre-cut with a batch processing manner with a specially designed software automatically. The input images were uniformly resized to 224 × 224 pixels, which is a standard-setting for deep learning methods. Each pixel’s RGB values of a photograph were normalized in a range from 0 to 1.

### Establishment of different DL-based diagnostic models of BK

**Figure 1:**
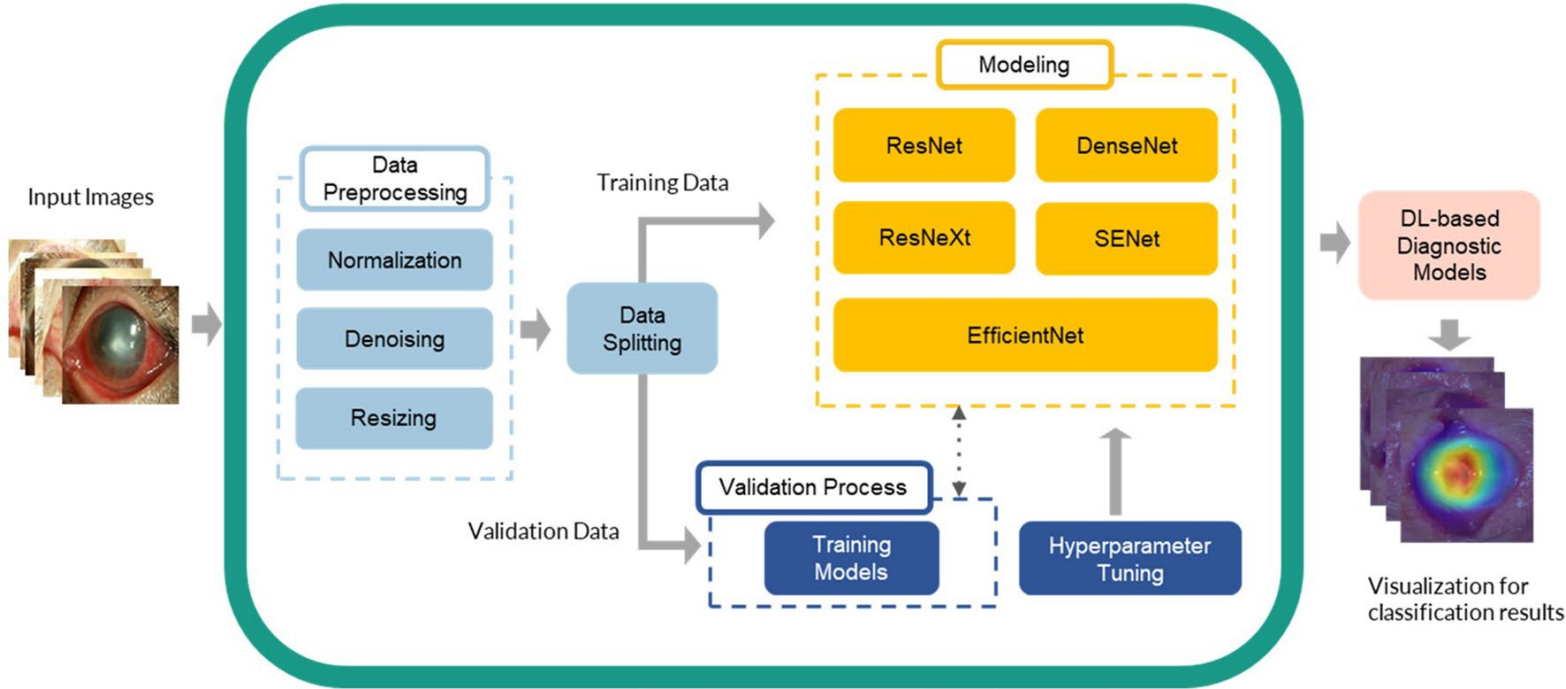
The framework of various deep learning models for diagnosing bacterial keratitis by external eye photographs. The input images are split into two sets, training dataset and validation dataset. In the data preprocessing, we utilize denoising to remove the noisy regions on the images and normalize and resize the images for further modeling. ResNet, DenseNet, ResNeXt, SE-ResNet, and EfficientNets were adopted in the modeling phase. The validation process was used to verify their performance. Moreover, hyperparameter tuning was used to optimize all models. After the optimization, the well-trained deep learning models can generate the heatmaps to demonstrate the regions on images for the visual explanation. The figure was created with Microsoft PowerPoint 2016.

**Figure 2:**
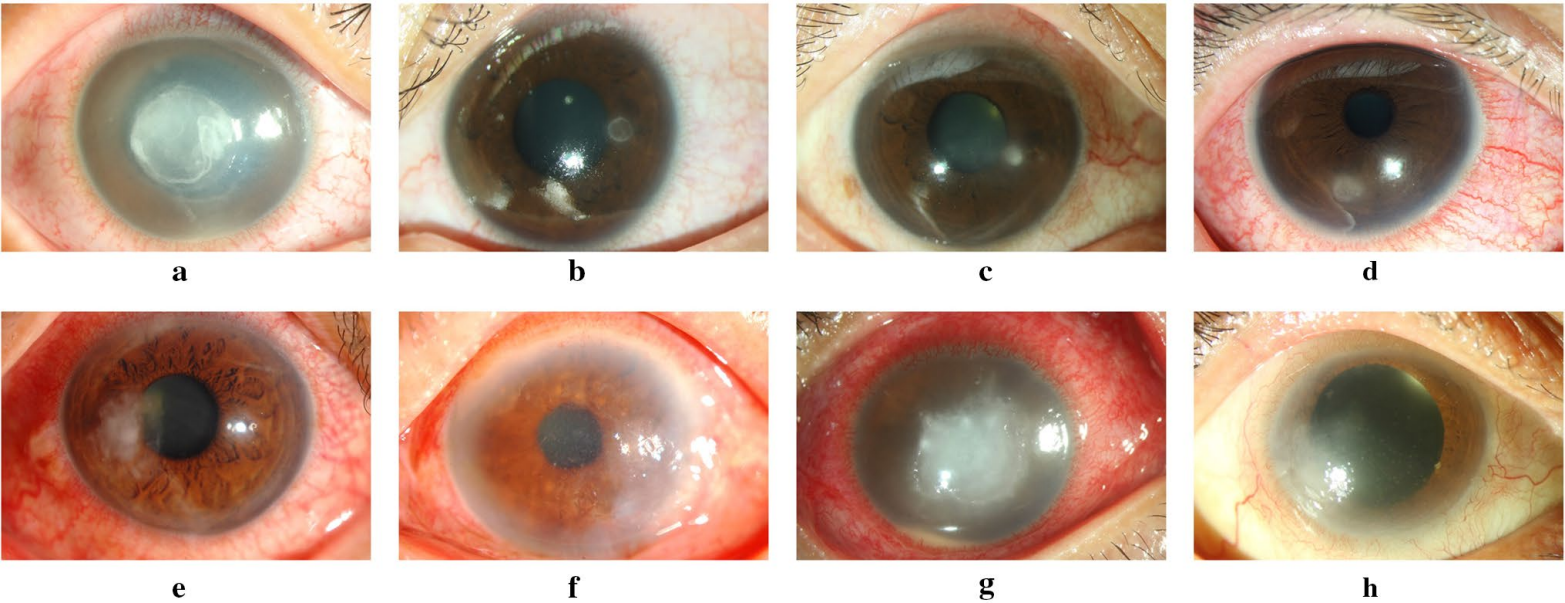
Representative photographs of microbial keratitis caused by bacterial and non-bacterial pathogens. Bacterial keratitis (**a**–**d**) and non-bacterial keratitis (**e**–**h**): (**a**) *Pseudomonas* keratitis, (**b**) *Serratia* keratitis, (**c**) non-tuberculosis *mycobacterium* keratitis, (**d**) *Streptococcus* keratitis, (**e**) *Fusarium* keratitis, (**f**) Herpes simplex keratitis, (**g**) *Acanthamoeba* keratitis, (**h**) Microsporidial stromal keratitis.

The framework shown in Fig. 1 was the newly established DL models for diagnosing BK via an external eye photo in this study. The training images were used to train a DL model for differentiating BK from non-BK photos (Fig. 2), whereas the validation images were used to test the performance of a trained model. After the randomization, each diagnostic model was trained with the respective DL algorithm toward the target with the greatest area under the receiver operating characteristic curve (AUROC). To generate the optimal model, we empirically tuned the hyperparameters of each model, including learning rate, the number of dense blocks, growth rate, and batch size according to the validation results. The Grad-CAM++ was applied for a visual explanation of these DL models^22^. The models were implemented in PyTorch, and all the experiments were performed on NVIDIA GeForce RTX 1080 GPUs.

### Diagnostic validation

Five-fold cross-validation was adopted to determine the sensitivity, specificity, positive predictive value (PPV), negative predictive value (NPV), and accuracy of each DL diagnostic model. In brief, the photos were classified as BK group (n = 929) and non-BK group (n = 583), which included FK (n = 383), VK (n = 128), and PK (n = 72). The photos of each group were randomly and equally assigned into five datasets (stratified fivefold cross-validation). There were 185–186 photos of BK & 116–117 photos of non-BK in each dataset. Four of the five datasets were used to train a DL diagnostic model, and the residual one was used to validate the model. Thus, there were five rounds of experiments for the performance validation of DL models.

### Statistical analysis

The average sensitivity, specificity, PPV, NPV, and accuracy of diagnosing BK were compared for different DL models. The 95% Wilson/Brown binomial confidence intervals for the above indices were estimated. The Fisher’s exact test was used for pairwise comparison of the performance index between two different DL models. Moreover, AUROC was alternatively used to compare the performance of various DL models, and the Z score test determined the statistical difference between any two models. A significant difference was set at *P* < 0.05 and analyzed by GraphPad Prism version 9.2.0 for Windows (GraphPad Software, San Diego, CA).

## Results

### Performance of the non-EfficientNet DL models for diagnosing BK

The average performances of five cross-validations of the four non-Efficient DL models were shown in Table 1. Among the four DL models, SE-ResNet50 revealed the highest sensitivity (82.4%), PPV (74.4%), NPV (66.5%), and accuracy (71/7%), while ResNeXt50 showed the highest specificity (55.1%). However, all the performance indices for diagnosing BK did not reach statistical difference between any two models in the four non-EfficientNet DL models.

**Table 1:** Diagnostic performance of non-EfficientNet deep learning models for diagnosing bacterial keratitis.

Model	Diagnostic performance (95% confidence interval)				
	Sensitivity	Specificity	PPV	NPV	Accuracy
ResNet50	81.1	50.4	72.3	62.7	69.3
	(74.2−87.8)	(41.7−59.1)	(68.6−75.9)	(52.1−73.3)	(63.9−74.5)
ResNeXt50	79.1	55.1	73.8	62.4	69.8
	(71.8−86.3)	(44.1−65.9)	(68.1−79.3)	(51.6−73.2)	(62.8−76.8)
DenseNet121	81.2	53.2	73.4	64.2	70.4
	(74.2−88.0)	(46.8−59.4)	(71.5−75.3)	(57.1−71.3)	(67.3−73.4)
SE-ResNet50	82.4	54.7	74.4	66.5	71.7
	(74.4−90.2)	(47.0−62.4)	(71.8−76.8)	(57.5−75.3)	(68.3−75.0)

### Performance of the EfficientNet DL models for identifying BK

The average diagnostic performances of the four Efficient DL models were shown in Table 2. Among the four DL models, EfficientNet B0 had the highest sensitivity (74.4%), whereas EfficientNet B3 had the highest specificity (64.3%) and PPV (76.8%). EfficientNets B1 and B3 had the highest NPV (61.1%) and accuracy (70.3%), equivalently. However, there was no significant performance difference between any two models in the four EfficientNet DL models in diagnosing BK.

**Table 2:** Diagnostic performance of EfficientNet deep learning models for diagnosing bacterial keratitis.

Model	Diagnostic performance (95% confidence interval)				
	Sensitivity	Specificity	PPV	NPV	Accuracy
EfficientNet B0	74.4	59.9	74.7	59.5	68.8
	(68.4−80.2)	(53.2−66.4)	(70.5−78.8)	(52.2−66.8)	(63.3−74.2)
EfficientNet B1	74.2	64.2	76.7	61.1	70.3
	(66.7−81.5)	(59.0−69.3)	(73.9−79.5)	(54.2−67.9)	(65.8−74.8)
EfficientNet B2	73.5	63.5	76.3	60.1	69.6
	(69.3−77.7)	(56.7−70.2)	(72.7−79.7)	(55.3−64.8)	(65.9−73.3)
EfficientNet B3	74.1	64.3	76.8	61.1	70.3
	(64.6−83.5)	(58.1−70.5)	(73.5−80.0)	(52.9−69.3)	(64.6−75.9)

### Comparing the EfficientNet models from the non-EfficientNet models in diagnosing BK

**Figure 3:**
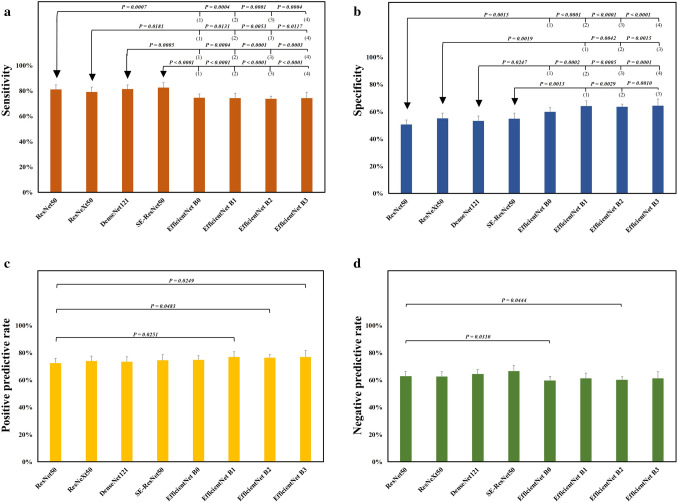
Comparison of the performance of various deep learning models via an external eye photo for diagnosing bacterial keratitis. (**a**) Diagnostic sensitivity of different deep learning models (**b**) Diagnostic specificity of the deep learning models (**c**) Positive predictive rate of the deep learning models (**d**) Negative predictive rate of the deep learning models. The arrow indicates a comparison between the pointed deep learning model and the model with a parenthesized number. *P* < .05 was recognized as a statistical difference and determined by the two-tailed Fisher’s exact test. The figure was produced by Microsoft Excel 2016 and PowerPoint 2016.

When comparing the four non-EfficientNet models (Table 1) and the EfficientNet models (Table 2), all non-EfficientNet models had significantly higher sensitivity than those of the EfficientNet models (Fig. 3a). In contrast, all EfficientNet models had significantly higher specificities than those of the non-EfficientNet models except for EfficientNet B0, which did not reach significance when compared with ResNeXt50 or SE-ResNet50 (Fig. 3b). EfficientNets B1, B2, and B3 models had significantly higher PPV than that of the ResNet50 (Fig. 3c), whereas the ResNet50 had significantly higher NPV than those of the EfficientNets B0 and B2 (Fig. 3d).

**Figure 4:**
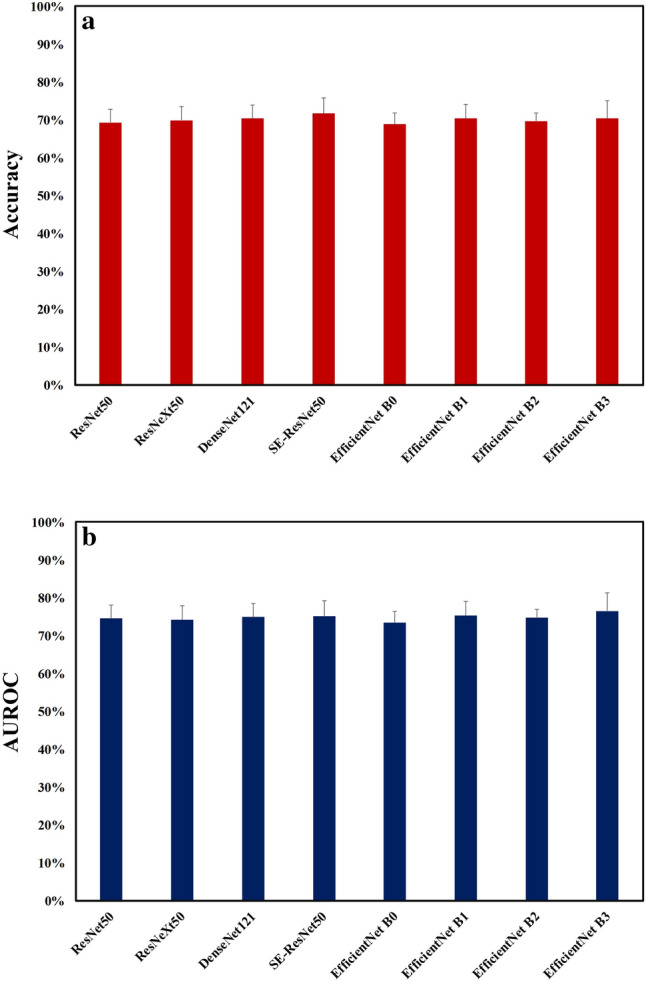
Comparison of the accuracy and area under the receiver operating characteristic curve of various deep learning models via an external eye photo for diagnosing bacterial keratitis. (**a**) Diagnostic accuracy of different deep learning algorithms (**b**) Area under the receiver operating characteristic curve of various deep learning models. AUC, the area under the receiver operating characteristic curve. The figure was produced by Microsoft Excel 2016 and PowerPoint 2016.

**Figure 5:**
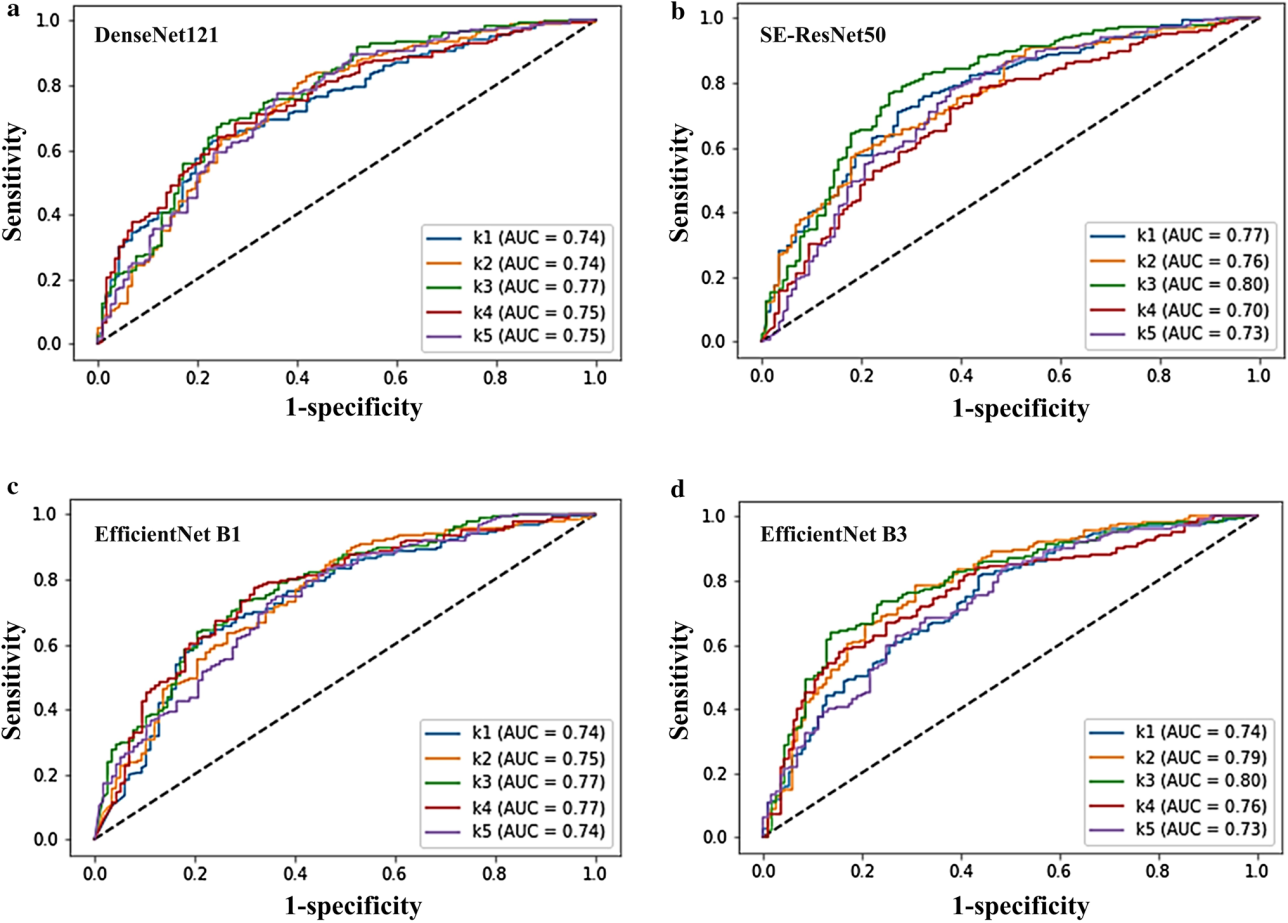
Receiver operating characteristic curves obtained from the fivefold cross-validation of the four best diagnostic models. (**a**) Receiver operating characteristic (ROC) curves of the DenseNet121 model (**b**) ROC curves of the SE-ResNet50 model (**c**) ROC curves of the EfficientNet B1 model (**d**) ROC curves of the EfficientNet B3 model. k1 to k5 represents the obtained ROC curves from the five test results of fivefold cross-validation. AUC, the area under the ROC curve. The ROC curve was plotted with the machine learning library, scikit-learn (https://scikit-learn.org/stable/auto_examples/model_selection/plot_roc.html#sphx-glr-auto-examples-model-selection-plot-roc-py), and with Python 3.7 for implementation.

The accuracy and AUROC summarize the above performance indices in diagnosing BK. We found that all non-EfficientNet and EfficientNet models had no significant difference in diagnostic accuracy (ranged from 68.8% to 71.7%; Fig. 4a) and AUROC (ranged from 73.4% to 76.5%, Fig. 4b). The receiver operating characteristic curves of the fivefold cross-validation of the four models with the greatest AUROCs, SE-ResNet50, DenseNet121, EfficientNets B1, and B3, were shown in Fig. 5.

## Discussion

BK is the most common IK in subtropical regions^2,23^ and is a principal cause of corneal scar leading to visual loss worldwide^3^. Recently, some authors reported that DL-based image diagnosis had an excellent diagnostic rate for BK^15,16,24^. Their results showed that different DL algorithms possessed diverse diagnostic performance, in which DenseNet and ResNet50 revealed the best performance^15,16,24^. However, these researchers adopted different niches to promote their own DL models in diagnosing BK, which made the actual performance of DL models incomparable among these studies. Therefore, we compared the potential DL algorithms via an external eye photo under the same verification setting without adding a fluorescence staining photo, performing image segmentation, or transforming the images before running a DL model for diagnosing BK. This study found non-EfficientNet models (ResNet50, ResNeXt50, DenseNet121, SE-ResNet50) were more sensitive than EfficientNet models (EfficientNets B0, B1, B2, and B3), while EfficientNet models were more specific than non-EfficientNet models. All the above models had comparable accuracy and AUROC.

In this study, not all IK were confirmed by laboratory tests. The confirmation rate of BK, FK, VK, and PK was 54%, 68%, 23%, and 47%, respectively. According to their clinical presentations and treatment histories, some subjects were diagnosed unanimously by the three corneal experts. Most BK and FK patients had typical presentations for these subjects, and they were treated early and successfully with empirical regimens, making laboratory tests unnecessary or unrecovered. Most VK patients were herpes keratitis, and most PK subjects were microsporidial keratitis. The epithelial type of herpes and microsporidia keratitis was usually diagnosed by the pathognomonic signs and response to treatment. Therefore, we must incorporate experts’ consensus diagnosis as the supplementary diagnostic standard to include these typical subjects. Unavoidably, there may be few subjects inherently misclassified into other kinds of IK. However, many DL models adopted experts’ diagnosis or grading as a gold standard^25–27^, and a DL system learning from experts’ impressions may decrease the interference from atypical presentations of some IK subjects. Thus, we ultimately decided to include the subjects with consensus diagnosis from three experts in this study.

The photographic diagnosis of BK via ophthalmologists was reported with 66–75% sensitivity and 68–90% specificity^28,29^. In our study, the sensitivity and specificity of the four non-EfficientNet models were 79–82% and 50–55%, respectively. The sensitivity and specificity of the EfficientNet models were 73–74% and 60–64%, respectively. These DL models had higher sensitivity but lower specificity than those of the ophthalmologists in the image diagnosis of BK. Redd et al. adopted a pre-trained ResNet50 model and used 70 test images, and they found the sensitivity and specificity in diagnosing BK was about 70% and 80%, respectively^24^. Under the same image-level as our study, which had no assistant processing such as segmentation, the accuracies of DL models VGG-16, GoogLeNet-v3, and DenseNet were 48.8%, 53.4%, and 60.5%, respectively^15^. Hung et al. did not show the data but mentioned nearly a 70% accuracy in diagnosing BK via a DenseNet model with a combination of two image types (external eye photos and fluorescence staining photos) under no image segmentation processing^16^. In our study, we found the accuracy of candidate models was 68.8–71.7% (Tables 1 and 2), which was higher than the best result (60.5%) of Xu et al. in the same image level^15^.

We further adopted AUROC as a performance index to compare these potential DL algorithms in diagnosing BK (Fig. 4b). The top four models based on AUROC were EfficientNet B3, EfficientNet B1, SE-ResNet50, and DenseNet121 in order. SE-ResNet incorporates the channel-attention operation of SENet to booster ResNet by focusing on learning the crucial channels^10^, and DenseNet can lessen the vanishing-gradient problem, fortify feature propagation, encourage feature reuse, and considerably reduce the number of parameters^8^. EfficientNet architectures are upgraded from baseline B0 (lowest computation cost) to highest B7 (highest computation cost with theoretically best accuracy)^11^. Due to the limitation of our computation resource, this study used EfficientNet-B0 to B3 models. Therefore, we can expect a growing performance in diagnosing BK via an external eye photo to be achieved by incorporating different DL models or introducing a more effective DL model.

In addition to introducing more powerful DL algorithms, there are other potential methods for promoting a DL model’s performance in diagnosing BK via external eye images. Xu et al. introduced a sequential-level feature learning model by annotating the centroid of the lesion to build a minimum circumscribed area and then partitioning the scaling up circular rings for training^15^. They found that this approach promotes the accuracy of diagnosing BK from 60.5% (image level via DenseNet) to 75.3% (sequential level via random-ordered patches). Hung et al. reported their DL diagnostic system for BK achieved 80% to 96% accuracy^16^. They adopted two images (external eye photos and fluorescence staining photos) and developed an additional image segmentation model for obtaining a cropped cornea before running a DL model. In addition, they narrowed the classification targets, which included only images of BK and FK for identification. Redd et al. adopted a specially designed portable digital camera with a pre-trained ResNet50 model^24^. They used 70 photos (50% were BK and 50% were FK) for testing and found that the diagnostic accuracy for BK was 76%. This study implied pictures from the same photographic system might promote the performance of a DL model. All of the above approaches showed that the image-based DL technology is an up-and-coming tool for diagnosing BK.

**Figure 6:**
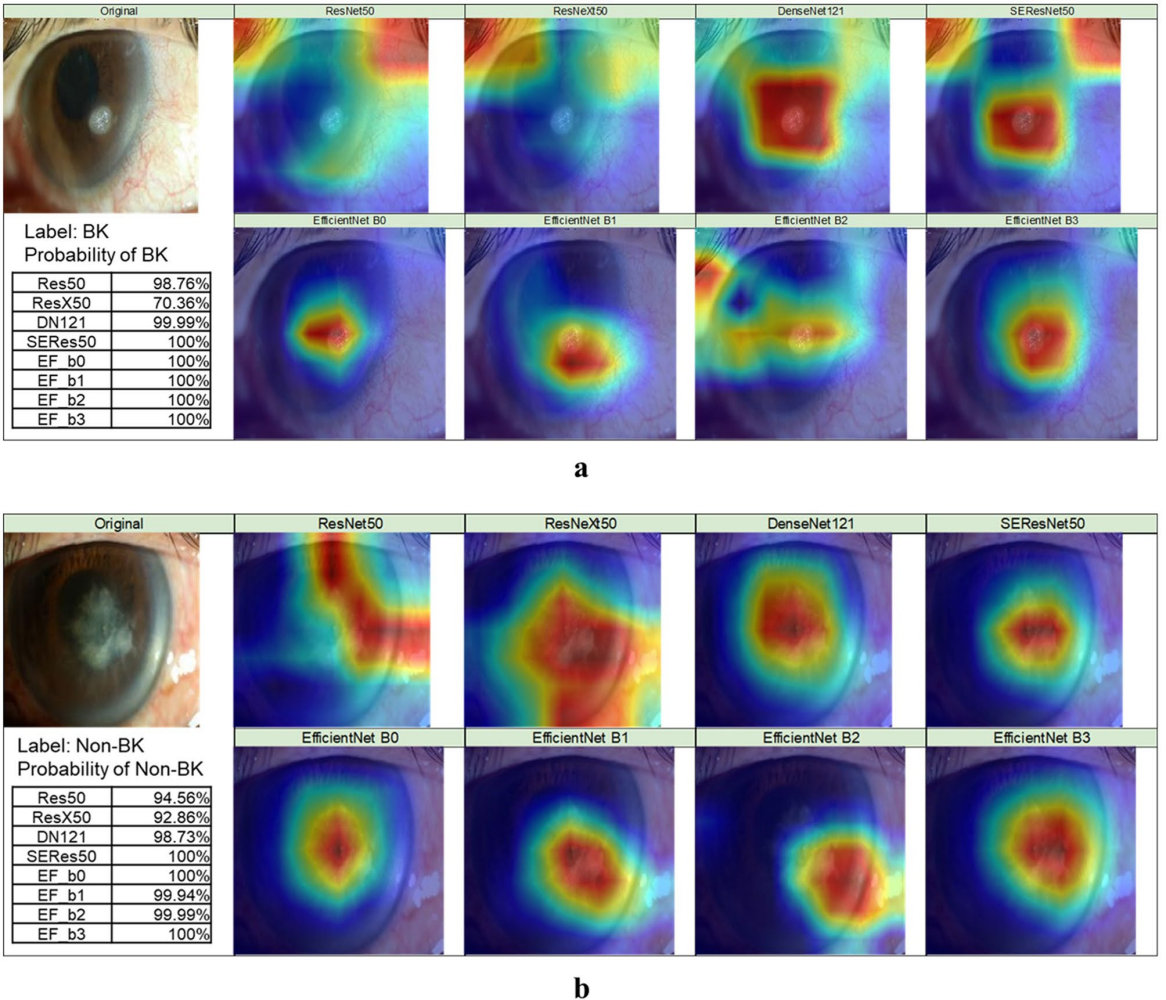
The classification results from different DL models with heat maps. (**a**) The probability of BK for each DL model and their heat maps. (**b**) The probability of Others for each DL model and their heat maps. The heat maps were created with Grad-CAM++ algorithm^22^, which was implemented with Python 3.7 (URL: https://www.python.org/downloads/release/python-370/).

In this study, we used Grad-CAM++ to generate the heat maps for explaining the results from DL models. In Fig. 6a, although all DL models correctly classified a BK image, the distribution of the heat maps demonstrated that EfficientNets were more focused on the lesions. The other models may have focused on not only the correct regions but also loci that were out of the lesions. In Fig. 6b, the classification results of a non-BK image showed that most models focused on the lesions, though ResNet50 and ResNeXt50 covered a more extensive range.

In conclusion, it is practical to promote the performance of an AI system for image diagnosis of BK via adopting a robust DL algorithm. SE-ResNet, DenseNet121, EfficientNets B1 and B3 possessed the greatest AUROC, in which DenseNet was recognized as the best DL algorithm in diagnosing not only BK but also FK^15,16,30^. We believe the requirement of an additional fluorescence staining photo, sophisticated image segmentation or transformation, and a specially designed camera will be gradually decreased by introducing a more effective DL model in diagnosing BK based solely on an external eye image. This approach may be more practical and useful in clinical settings without ophthalmological medical personnel.
